# Sensorimotor Contingencies as a Key Drive of Development: From Babies to Robots

**DOI:** 10.3389/fnbot.2019.00098

**Published:** 2019-12-04

**Authors:** Lisa Jacquey, Gianluca Baldassarre, Vieri Giuliano Santucci, J. Kevin O’Regan

**Affiliations:** ^1^Integrative Neuroscience and Cognition Center, UMR 8002, CNRS, Université Paris Descartes, Paris, France; ^2^Laboratoire Ethologie Cognition Développement, Université Paris Nanterre, Nanterre, France; ^3^Laboratory of Computational Embodied Neuroscience, Istituto di Scienze e Tecnologie della Cognizione, Consiglio Nazionale delle Ricerche, Rome, Italy

**Keywords:** infant development, sensorimotor development, sensorimotor contingencies, goals, unsupervised learning

## Abstract

Much current work in robotics focuses on the development of robots capable of autonomous unsupervised learning. An essential prerequisite for such learning to be possible is that the agent should be sensitive to the link between its actions and the consequences of its actions, called sensorimotor contingencies. This sensitivity, and more particularly its role as a key drive of development, has been widely studied by developmental psychologists. However, the results of these studies may not necessarily be accessible or intelligible to roboticians. In this paper, we review the main experimental data demonstrating the role of sensitivity to sensorimotor contingencies in infants’ acquisition of four fundamental motor and cognitive abilities: body knowledge, memory, generalization, and goal-directedness. We relate this data from developmental psychology to work in robotics, highlighting the links between these two domains of research. In the last part of the article we present a blueprint architecture demonstrating how exploitation of sensitivity to sensorimotor contingencies, combined with the notion of “goal,” allows an agent to develop new sensorimotor skills. This architecture can be used to guide the design of specific computational models, and also to possibly envisage new empirical experiments.

## Introduction

Over recent years there has been growing interest in constructing robotic systems whose physical and cognitive competence evolves progressively in a way analogous to how biological systems, and in particular humans, develop in the womb and after birth. A variety of approaches to such unsupervised learning are currently being investigated (e.g., cf. Baldassarre and Mirolli, 2013; Aubret et al., 2019) and all of them share an underlying assumption, which is that one of the main mechanisms agents use to learn about themselves and their environments consists in making exploratory actions, and then evaluating the effects of those actions on their sensory input. The laws that govern such relations between actions and resulting sensory changes have sometimes been called “sensorimotor contingencies” (MacKay, 1967; O’Regan, 2011). In robotics it is taken for granted that agents must be sensitive to sensorimotor contingencies, and research focuses on the details of how this sensitivity allows new cognitive, motor, and social skills to be acquired.

In developmental psychology it is also assumed that sensorimotor contingencies are an important driver to learning. Some research has been done to evaluate the infant’s basic sensitivity to such contingencies. For example, researchers have demonstrated that sensitivity to sensorimotor contingencies is already present in fetuses and newborns for stereotyped actions such as hand-mouth coordination (e.g., Myowa-Yamakoshi and Takeshita, 2006) or non-nutritive sucking (e.g., DeCasper and Spence, 1986), and then that it extends to other actions in the first months of life (e.g., Rovee and Rovee, 1969). Some research has also been done on the very basic question of the timing of sensorimotor contingencies (e.g., Ramey and Ourth, 1971; Millar, 1972; Zmyj et al., 2009). Another avenue of research has focused on how in the very first stages of development the infant can use sensorimotor contingencies to distinguish what it has systematic control over from what it only has partial control over. This distinction presumably allows the infant, respectively, to determine what is likely to be part of its own body versus what is part of the outside world (e.g., Gergely and Watson, 1999; Butko and Movellan, 2010; Terekhov and O’Regan, 2016). Here however we shall be focusing on the much greater volume of research in developmental psychology devoted to documenting how, after these beginnings, the infant goes on to use sensorimotor contingencies to understand the structure of its own body, its sensory systems, and also its proximal and distal environment, including the social environment. Ultimately, it is assumed that sensitivity to sensorimotor contingencies will contribute to the establishment of socio-cognitive and motor skills (Gergely and Watson, 1996; Tarabulsy et al., 1996; Nagai and Asada, 2015; Verschoor and Hommel, 2017) like language (e.g., Goldstein and Schwade, 2008; Schwartz et al., 2012), reaching and grasping (e.g., Corbetta and Snapp-Childs, 2009) and walking (e.g., Adolph et al., 2000).

Our article tries to make the connection between this quite extensive literature in developmental psychology and current robotic approaches to motor and cognitive development. It is hoped that results from experiments with infants might provide inspiration to roboticists, and, conversely, we hope that existing robotic models and algorithms could perhaps be invoked by developmentalists to explain their data and to stimulate new experiments. We chose to focus on four questions as examples of where cross-fertilization might occur between developmental psychology and robotics: (1) Are infants able to identify the particular motor skills or body parts involved in a sensorimotor contingency? (2) How long is a sensorimotor contingency retained in memory? (3) To what extent are infants capable of generalizing a sensorimotor contingency to a new situation? (4) Are infants able to use sensorimotor contingencies in order to attain desired states?

In what follows we will look at how these four questions have been experimentally addressed in developmental psychology — for reference, experimental methods and results of the studies reviewed in this paper are summarized in a table for each section (respectively Tables 1–4). In each section, we will link infant data to the main computational and developmental robotics literature also reviewed in a prospective and critical way. Note that the “Approaches in robotics” sections do not claim to be exhaustive, but suggest possible links between infant study findings and current work in developmental robotics. We close the paper by presenting a blueprint architecture integrating the key elements of contingency-based learning from a robotics perspective. This can be used to guide the design of specific computational models and also to possibly envisage new empirical experiments (the architecture was introduced in Baldassarre et al., 2018).

**TABLE 1:** Summary of infant studies discussed in the section “Body knowledge”.

**Paper**	**Age**	**Paradigm**	**Main conclusion**
**Motor skill refinement**			
Thelen, 1994; Angulo-Kinzler, 2001; Angulo-Kinzler et al., 2002; Chen et al., 2002; Tiernan and Angulo-Barroso, 2008; Sargent et al., 2014, 2015	3–4 months	Mobile paradigm involving a task-specific (unusual) leg movement	Contingency learning could lead infants to explore task-specific (unusual) actions.
Needham et al., 2002	3 months	Sticky mittens	Sensorimotor experience of reaching could help infants to develop manual exploration.
Lee and Newell, 2013	3–4 months	Operant conditioning between arms movements and auditory feedback	Contingency training could help infants to develop manual exploration.
Needham et al., 2014	3 months	Reinforcement (mobile paradigm) of reaching movements	Contingent feedback could facilitate the acquisition of reaching.
Williams and Corbetta, 2016	3 months	Longitudinal training for reaching with or without feedback	Contingency training could facilitate the acquisition of reaching.
**Body part differentiation**			
Rovee-Collier et al., 1978; Heathcock et al., 2005	3–4 months	Mobile paradigm (leg movement)	3- to 4-month-old infants seem able to narrow down a contingency to a specific limb.
Watanabe and Taga, 2006	2, 3, and 4 months	Mobile paradigm (arm movements)	Limb-specific response in a contingent task develops between 2 and 4 months.
Watanabe and Taga, 2009	3 months	Mobile paradigm (arm or leg movements)	Limb-specific response in a contingent task emerges for arms before legs.
Kato et al., 2013	2 and 3 months	Mobile paradigm (arm movements)	Inhibition necessary for a limb-specific response develops between 2 and 3 months.
D’Souza et al., 2016	9 and 12 months	Rattle shaking	Extraneous “overflow” movements decrease with age.
Begum Ali et al., 2015	4 and 6 months	Buzzer location (feet)	From 4 months of age infants seem able to implicitly localize a buzzer.
Bremner et al., 2008	6.5 and 10 months	Buzzer localization (hands)	Infants’ ability to visually localize a buzzer improves during development.
Hoffmann et al., 2017	3 to 12 months	Buzzer localization (body)	Infants’ ability to reach a buzzer improves during development.
Somogyi et al., 2018	3, 4, 5, and 6 months	Buzzer localization (body)	Infants’ response to a buzzer becomes more localized during development.
Chinn et al., 2019	3 to 12 months	Buzzer localization (body)	Infants’ ability to reach a buzzer improves during development.

**TABLE 2:** Summary of infant studies discussed in the section “Memory”.

**Paper**	**Age**	**Paradigm**	**Main conclusion**
**Retention**			
Rovee-Collier, 1999 review	2 to 18 months	Mobile paradigm Train paradigm	Infants’ contingency retention capacity improves during development: 1 day at 2 months, 1 week at 3 months, and 3 months at 18 months.
**Reactivation**			
Fagen and Rovee-Collier, 1983	3 months	Mobile paradigm	Reactivation restores the memory of a contingency even when babies no longer show any sign of retention of this contingency.
Rovee-Collier et al., 1981	3 months	Mobile paradigm	Temporal course of forgetting of a contingency after reactivation is similar to the one of a newly acquired contingency.
Rovee-Collier, 1999 review	2 to 18 months	Mobile paradigm Train paradigm	The time interval after which the reactivation treatment becomes effective decreases during development: 24 h at 2 months and immediately at 12 months and older.
Rovee-Collier, 1999 review	2 to 18 months	Mobile paradigm Train paradigm	The exposure time to the treatment required for it to be effective decreases during development: 2 min at 3 months and 1.8 s at 12 months.
Rovee-Collier et al., 1980	3 months	Mobile paradigm	Reactivation does not require practicing the contingency, but only perception of the stimulus involved in the contingency.

**TABLE 3:** Summary of infant studies discussed in the section “Generalization”.

**Paper**	**Age**	**Paradigm**	**Main conclusion**
**Change in stimulus**			
Milewski and Siqueland, 1975	1 month	Non-nutritive sucking	From very early on, infants can generalize their learning of a contingency to a new stimulus when the change occurs immediately after learning.
Hartshorn et al., 1998 review Rovee-Collier, 1999 review	2 to 12 months	Mobile paradigm Train paradigm	Before 6 months, infants cannot generalize their learning of a contingency to a new stimulus when the change occurs 1 day after learning. After 6 months, infants can generalize their learning of a contingency to a new stimulus when the change occurs within 2 weeks, but not for longer delay.
Butler and Rovee-Collier, 1989	3 months	Mobile paradigm	Young infants cannot generalize their learning of a contingency to a new stimulus and context when the change occurs 1 day after learning.
Fagen and Rovee, 1976 Mast et al., 1980	3 months	Mobile paradigm	Young infants can generalize their learning of a contingency when quantitative aspects of feedback are modified immediately or 24 h after learning.
Fagen et al., 1976	3 months	Mobile paradigm	A change of more than two objects in the mobile prevents generalization.
Hayne et al., 1986	2 months	Mobile paradigm	A change of more than one object in the mobile prevents generalization.
Adler and Rovee-Collier, 1994	3 months	Mobile paradigm	A change in appearance of the elements of the mobile (painted letters) prevents generalization.
**Change in context**			
Hartshorn et al., 1998 review Rovee-Collier, 1999 review	2 to 12 months	Mobile paradigm Train paradigm	Infants are able to generalize their learning to a new crib when tested 1 day after training before 6 and up to one month after 6.
Hayne et al., 1991	3 months	Mobile paradigm	A change of room prevents generalization when testing occurs 2 weeks after training but not when it occurs 1 day after training.
Hartshorn and Rovee-Collier, 1997	6 months	Train paradigm	A change of room prevents generalization when testing occurs 3 weeks after training and is preceded by a reactivation the day before.
Rovee-Collier and Dufault, 1991	3 months	Mobile paradigm	Training in multiple contexts overrides the lack of generalization to a new context.

**TABLE 4:** Summary of infant studies discussed in the section “Goal-directedness”.

**Paper**	**Age**	**Paradigm**	**Main conclusion**
**Means-end behavior**			
Lobo and Galloway, 2008	5–6 months	Select a lever to obtain an effect	Around 5–6 months babies show early signs of means-end behaviors.
Munakata et al., 1997; Willatts, 1999; Babik et al., 2018	6 to 24 months	Pull a cloth to reach an object	7-month-old babies show means-end behaviors.
Rat-Fischer et al., 2014	16 to 20 months	Select a string to reach an object	Even in the second year of life, not all babies are able to select the right action to achieve a goal, here the string to pull to get a toy.
Carpenter et al., 2005	12 and 18 months	Action imitation	Infants copy the end or the means of an adult action depending on the context.
**Anticipation abilities**			
Wang et al., 2012	6 and 8 months	Gaze contingency (visual feedback)	Infants can anticipate the consequences of their actions, here the appearance of a picture following gaze movements.
Kenward, 2010	10 months	Button contingency (audiovisual feedback)	Infants can anticipate the consequences of their actions, here the appearance of a video after pushing a button.
**Bidirectionality of contingency learning**			
Verschoor et al., 2010	9, 12, and 18 months	Operant conditioning between touch and auditory feedback	Infants are faster to produce an action after occurrence of its previously learned effect than after occurrence of an unlinked stimuli.
Verschoor et al., 2013	7 and 12 months	Operant conditioning between eye movements and auditory feedback	After learning two different contingencies, 12-months-old babies are able to produce the action associated with each stimulus, but 7-month-old babies are not able to do so.
**“Representation” of the action-outcome coupling**			
Klossek et al., 2008	16 to 27, 27 to 37, and 37 to 48 months	Revaluation paradigm	Before the age of 2 years, babies are not able to select and carry out the action (between 2 actions) required to achieve a desired goal.
Kenward et al., 2009	14, 19, and 24 months	Revaluation paradigm	Before the age of 2 years, babies do not perform an action more frequently when it achieves a desired goal than when it triggers an unspecified effect.

We are aware that remarkable work has already linked data from developmental robotics and developmental psychology (e.g., Guerin et al., 2013; Caligiore et al., 2014a; Cangelosi and Schlesinger, 2015; Jamone et al., 2016; Oudeyer et al., 2016; Caligiore and Baldassarre, 2019). The novelty of our approach is to focus more precisely on how sensorimotor contingencies are exploited (since as such it has not been a main focus of developmental robotics) and to illustrate with four examples how this sensitivity contributes to development. In our survey, we shall concentrate on studies involving babies under 1 year of age, where presumably the mechanisms involved in contingency detection will be easier to study, since they will be less likely to be masked by higher learning processes.

Finally note that we are aware that our enterprise is very preliminary. Up until now, developmental psychologists have rarely used robotic models to inspire their experiments or to test their explanations. Conversely, roboticists have also not generally attempted to explain developmental data, but have simply used psychology as inspiration and adopted ideas from the developmental literature when this has helped them solve a problem. For this reason, the reader must not expect to find a very tight link between the developmental and the robotic sections in our paper. We have nevertheless attempted to make links when these are possible.

## Body Knowledge: Is the Agent Able to Identify the Particular Motor Skills or Body Parts Involved in a Sensorimotor Contingency?

In two seminal papers Barto et al. (2004) and Singh et al. (2004), proposed a simulated agent playing in the equivalent of a “baby gym,” learning how its actions modified input coming from the environment. By discovering increasingly complex sensorimotor contingencies, the agent was able to autonomously acquire different behaviors. Oudeyer et al. (2005) then implemented a robotic instance inspired by that work, where an AIBO robot in a baby playpen could discover both the functioning of its own body, and also the actions that its immediate environment afforded. Even though the agents of these experiments were limited in the number of sensors used and the number of actions they could perform, and even though the approach proposed was difficult to scale up to a truly open-ended environment, this scenario is interesting because it can be applied both to robots and to human babies with respect to the question of the acquisition of body knowledge (see Forestier et al., 2017; Mannella et al., 2018; and Nair et al., 2018, for recent examples of models able to autonomously learn multiple tasks and skills embedded in complex spaces). Thus, one can imagine that the agent starts by doing random “motor babbling.” At every moment it activates a variety of effectors and receives feedback through its sensors from the environment. For example, the agent might by chance touch a bell suspended near it, and perceive a resulting auditory input. Its “intrinsic motivation” might then prompt it to attempt to replicate the sound by repeating the action it performed. By progressively refining its actions, will the agent be able to precisely identify the motor command that produced the bell sound? Will this ultimately allow the agent to acquire that command as a new implicit skill, thereby contributing to better distinguish its body parts?

### Evidence From Developmental Psychology

#### Motor Skill Refinement

Researchers have investigated to what extent exploration of sensorimotor contingencies could lead infants to refine their motor skills through the use of the “mobile paradigm.” This paradigm, which was developed and intensively used with 3–4-month-old infants by Rovee-Collier and her collaborators, consists in using a ribbon to attach one limb (leg or arm) of the infant to a mobile placed above the infant, in such a way that movements of the “connected” limb of the infant produce contingent movement of the mobile. In order to study motor skill refinement, researchers also developed modified versions of the “mobile paradigm” in which leg movements which were not in the infant’s usual repertoire of actions generated the contingent feedback. For example, Angulo-Kinzler et al. (2002) tested 3-month-old infants and used knee extensions and knee flexions as triggering action. The authors showed that a few minutes after the beginning of exposure to the contingency, infants started to explore these non-usual leg movements. Interestingly, after a longer exposure, infants refined the necessary motor action and were able to make the actions in a more efficient manner. Similar observations were made in several other studies (e.g., Thelen, 1994; Angulo-Kinzler, 2001; Chen et al., 2002; Tiernan and Angulo-Barroso, 2008; Sargent et al., 2014, 2015), suggesting that infants’ exploration of sensorimotor contingencies might play a role in motor skill refinement. Developmental psychologists have also measured the influence of contingent feedback on the development of emerging skills such as reaching. The best-known example is the “sticky mittens” experiment (e.g., Needham et al., 2002) in which 3-month-old babies who are not yet capable of reaching/grasping received 2 weeks of daily training sessions during which they carried mittens with Velcro attached to their palm, allowing them to pick up objects. After this training, babies that had had this new sensorimotor experience showed more (visual and oral) object exploration compared to non-trained babies. In another experiment, Lee and Newell (2013) exposed 10 to 16-week-old infants biweekly to a contingency between movements of one arm and auditory feedback. They found that this longitudinal intervention did not influence the moment in development when reaching emerged. However, they observed that compared to a control group, infants exposed to the contingency made more exploratory and “goal-directed” movements. Compatible with this, Needham et al. (2014) showed that 3-month-old infants were able to increase their reaching-like movements when these movements were contingently reinforced (here through the mobile paradigm, see above for a description). This increase in reaching-like movements was observed during contingency exposure but also directly afterward, when infants participated in a free-play experiment with a rattle. These results have been confirmed in a recent study by Williams and Corbetta (2016). The authors trained 3-month-old infants to reach for an object every day for 2 weeks. They found that infants for whom reaching movements toward the object generated contingent movements of the object, contacted the object significantly more than the control groups for whom reaching movements did not produce the contingent feedback.

#### Body Part Differentiation

The differentiated use of the body observed in adults does not seem to be present in very young infants (e.g., Watanabe and Taga, 2006), suggesting this ability develops during early infancy. We assume here that this development is allowed by accumulation of implicit knowledge provided by the detection of sensorimotor contingencies. Thus, one might expect that in the youngest infants, sensorimotor contingencies would not be specific to individual body parts, but rather that they would involve global body motions. However, as infants grow older one might expect that sensorimotor contingencies would narrow down to individual body parts. It is difficult to directly prove that there is such a causal link between accumulation of implicit knowledge provided by the detection of sensorimotor contingencies and body part differentiation. Below, we present what evidence there is more generally concerning the progressive differentiation of body responses over development.

Differentiation of body responses has been investigated in infants between 2 and 4 months of age by measuring their ability to narrow down a contingency to a specific limb, for instance to the limb connected to the mobile in the mobile paradigm (see description above). Around 3 months of age, infants exposed to the mobile paradigm with one of their legs connected increase the movements of both of their legs and not the movements of their arms or even only the movements of their connected leg (e.g., Rovee-Collier et al., 1978; Heathcock et al., 2005). Going one step further, the team of Watanabe and Taga (Watanabe and Taga, 2006, 2009; Kato et al., 2013) investigated how differentiation of body responses evolves between 2 and 4 months of age, again using the mobile paradigm but connecting one arm of the infant to the mobile. Watanabe and Taga (2006) observed that 2-month-old infants increased activity of their four limbs equally, 3-month-old infants increased activity of both of their arms equally but did not increase the activity of their legs, and 4-month-old-infants increased the activity of their connected arm only. In another paper, Watanabe and Taga’s team (Watanabe and Taga, 2009) showed that the moment when differentiation in body responses emerges differs depending on the body part involved. They found that 3-month-old infants with one arm connected increased activity of their arms only, but that infants of the same age with one leg connected increased the activity of their whole body. Interestingly, they proposed that this difference in response specificity might be explained by the different brain regions which controlled arm and leg movements (respectively the thalamocortical network and the brainstem network). More precisely, the authors suggested that the absence of limb-specific movement observed in the leg condition could be explained by the fact that leg movements are predestined for walking which requires coordination between both legs and between legs and arms. In a subsequent paper, the authors suggested that infants’ ability to inhibit their arm movements to perform one-arm movement becomes established between 2 and 3 months of age (Kato et al., 2013). D’Souza et al. (2016) have added a further interesting result by testing older infants’ motor specialization during unimanual actions. More precisely, they measured the number of extraneous movements made by 9- and 12-month-old infants during play with a rattle. Here again, they found evidence for a progressive refinement of body knowledge, since extraneous “overflow” movements decreased with age.

Differentiation of body responses has also been investigated by measuring infants’ responses to vibrotactile stimulation (Bremner et al., 2008; Begum Ali et al., 2015; Hoffmann et al., 2017; Somogyi et al., 2018; Chinn et al., 2019). In these experiments, the authors applied a vibrotactile stimulation on the infant’s body for several seconds at different locations (not simultaneously but one location after the other) and measured the ability of the infant to localize the buzzer on a specific body part. More precisely, the authors measured both infants’ implicit localizations — which limb was moved first or which limb was more active during the stimulation — and explicit localizations — looks/touches/grasps toward the buzzer. The authors found a developmental pattern for tactile stimuli applied to the body: at 4 months of age infants seemed able to implicitly localize the tactile stimulation; at 5–6 months they became able to touch and/or grasp it and only after 6 months did they start to look at it. More interestingly for our purpose, Somogyi et al. (2018) found that the youngest infants’ reactions (3 months of age) involved global body motions — i.e., the youngest infants moved their whole bodies during stimulation — whereas older infants’ reactions (5–6 months of age) involved limb-specific body motions — i.e., older infants moved the stimulated limb more.

#### Conclusion From Developmental Psychology

The studies reviewed above suggest that exploration of sensorimotor contingencies allows infants to refine motor skills and to differentiate their body parts. Thus, in the first 2 months of life, detecting contingent actions involve general motor activity of the infant. Then, the third month of postnatal life appears to be a pivot in development: detected contingent actions become more integrated into the refinement of existing skills. This change at 3 months of age is also reflected in body differentiation: arms and legs seem more differentiated. One month later, body knowledge specialization seems to be even more effective, and limb differentiation seems to be achieved. Thus, body knowledge development, achieved through sensorimotor contingency detection, presumably brings infants to acquire a repertoire of well-defined actions, leading them to be ready at 3–4 months of age to develop reaching, grasping and later, tool-use abilities. Another point is worth noting: gradual maturation of inhibitory processes might also be involved in the progressive specialization of motor responses. The lack of inhibition in motor babbling may be a factor that prevents younger infants from easily making limb-specific actions.^1^ It may be the increase in inhibitory abilities that leads older infants to discover limb-specific actions.

### Approaches in Robotics

Work on open-ended learning (Baldassarre and Mirolli, 2013) confirms that a simple agent equipped with what has been called “intelligent adaptive curiosity” can indeed acquire information about the effects that it can generate in the environment and leverage these sensorimotor contingencies to learn or refine its skills. Following the idea that understanding one’s effects on the environment is crucial for the autonomous development of animals and humans (White, 1959; Berlyne, 1960) different work in robotics has focused on the autonomous learning of skills on the basis of the interactions between the body of the artificial agent and the environment, where robots are tested in “simple” reaching or avoidance scenarios (e.g., Santucci et al., 2014; Hafez et al., 2017; Hester and Stone, 2017; Reinhart, 2017; Tanneberg et al., 2019) or in more complex tasks involving interactions between objects (da Silva et al., 2014; Seepanomwan et al., 2017), tool use or hierarchical skill learning (Forestier et al., 2017; Colas et al., 2018; Santucci et al., 2019), and even in imitation learning experiments (Duminy et al., 2018). When combined with the use of “goals,” intended here as specific states or effects that a system is trying to attain, curiosity and intrinsic motivation are able to properly guide task selection (Merrick, 2012; Santucci et al., 2016) and reduce the exploration space (Rolf et al., 2010; Baranes and Oudeyer, 2013).

On the other hand, the relation between sensorimotor contingencies and the development of early body knowledge *per se* is still poorly studied in robotics. Only few studies have used computational models to investigate the differentiation of body parts. Olsson and colleagues presented models where sensors and actuators were seen as information sources, thus using tools from information theory to help distinguish between them on the basis of the quantity and typology of information they provide (Olsson et al., 2004, 2006). In particular, the distance between two information channels allowed to infer the topology of sensors and motors in an AIBO robot. Looking at the formation of body maps, Hoffmann and colleagues in different studies have underlined how the development of the body schema may improve the functioning of artificial agents (Hoffmann et al., 2010; Roncone et al., 2014), as well as how robots can be used to model early sensorimotor development (Hoffmann et al., 2017; Hoffmann and Pfeifer, 2018). The way peripersonal motor maps can help robot learning has been studied in Jamone et al. (2014). Mannella et al. (2018) presented a specific hypothesis on the learning of sensorimotor contingencies in relation to the gradual acquisition of the knowledge of the agent’s own body driven by intrinsic motivations. The blueprint architecture introduced in the final section of this article describes this model more fully. Similarly, Juett and Kuipers (2019), using a Baxter robotic platform, study how visual sensorimotor contingencies are a key element in developing the robot’s knowledge of its own body and the way it relates to the environment.

The robotic approaches described above raise an important issue that might provide impetus for empirical investigations in developmental studies: given that we assume that sensitivity to sensorimotor contingencies is a driving force behind learning, the question arises of which of many possible contingencies should be considered as interesting and relevant to guide learning. The adaptive curiosity framework has proven the power of novelty and surprise in selecting which contingencies should be attended to, but it is not clear which events should be considered novel or surprising. State-of-the-art curiosity-driven robots are normally tested in controlled environments (whether in simulation or in laboratory) where all the interactions can generate fruitful learning processes. But in real-life scenarios, such robots would be stuck in focusing on every little “novel” or “unexpected” change perceived by their sensors, thus wasting a lot of time in useless or redundant activities. To cope with this limitation, it is necessary to build new models that can “evaluate” sensorimotor contingencies and decide which are worth triggering the learning processes and, from a psychological point of view, to create experiments that suggest which kind of mechanisms might be related to this evaluation process.

## Memory: How Long Is a Sensorimotor Contingency Retained in Memory?

Returning to the example of the agent in a baby gym (Barto et al., 2004; Singh et al., 2004; Oudeyer et al., 2005), let us imagine that yesterday it discovered a new contingency: for example, touching a bell to generate a sound. When returning to the baby gym today, will the agent remember what it learned yesterday, or must it relearn the contingency from scratch? And how does this depend on the delay since first encountering the contingency? How does practice in the interim affect recall?

### Evidence From Developmental Psychology

Rovee-Collier and her team are the authors who have contributed extensively to the question of memory of sensorimotor contingencies. Their studies involved infants from 2 to 18 months of age in an experimental framework consisting in training infants for 2 or 3 days with a sensorimotor contingency that involved 6 to 9 min of exposure on each training session, and then in testing infants’ retrieval of this contingency after different delays: from 1 day to a few months after training ended. In addition, in some of their studies they reactivated the learning between training and testing through a brief exposure to the contingent stimulus or to the context in which learning occurred. The paradigm they used was the mobile paradigm with infants aged less than 6 months, and the “train” paradigm with older infants — in this paradigm, the infant pushes on a button to trigger the motion of an electric train.

#### Retention

One of Rovee-Collier and her colleagues’ lines of research was the retention of sensorimotor contingencies (e.g., Sullivan et al., 1979). Rovee-Collier and her colleagues showed that 1 day after training with the mobile, infants of all ages were able to retrieve the contingency. However, for longer delays, the retrieval was dependent on the age of the infant: the duration of retention increased progressively from 1 day to 3 months, as the age of the infant increased from 2 to 18 months of age (see Rovee-Collier, 1999 for a review). It is worth noting that infants’ retention can be modulated by the training schedule. For example, for the same amount of training — 18 min — 2-month-old infants’ retrieval of a contingency increased from 1 day to 2 weeks when the training was spread over 3 days instead of 2 days (Rovee-Collier, 1999).

#### Reactivation

In another series of experiments, Rovee-Collier and her colleagues introduced a “reactivation treatment” (priming) 1 week (or two) after the last training session, when the infant had forgotten the contingency. For example, in Fagen and Rovee-Collier (1983), a reactivation treatment was applied 13 days after the last training session, the authors having previously shown that 3-month-old infants trained with the mobile paradigm showed no retention of the contingency when tested 14 days after the end of training (e.g., Sullivan et al., 1979). Moreover, the authors confirmed that 3-month-old infants showed no retention of the mobile contingency 2 weeks after training ends by including a control group tested 14 days after the last training session without reactivation and who showed no retention. The reactivation consisted in 3 min of exposure to the original mobile/train or to the original environment used previously (e.g., testing room, color of the infant’s crib). Rovee-collier and her team found that both these modes of reactivation enabled infants aged 3 months and older to again retrieve the forgotten contingency. The authors found that the temporal course of forgetting after reactivation was similar to the temporal course after training: forgetting increases with delay (e.g., Rovee-Collier et al., 1981). Moreover, the reactivation treatment becomes effective more quickly as age increases: while young infants (3 months of age) need around 24 h between reactivation and testing for the reactivation treatment to become effective, it becomes effective immediately for infants that are 12-months-old or older (Rovee-Collier, 1999). The delay needed to obtain this reactivation increases logarithmically over the first year of life. Also, the time of exposure needed to obtain reactivation decreases, going from 2 min at 3 months of age to 1.8 s at 12 months (Rovee-Collier, 1999).

#### Conclusion From Developmental Psychology

Take home messages from these studies are that (i) from very early in life (2 months of age), babies are able to remember a contingency 1 day after exposure, (ii) the duration of retention increases gradually to as much as 3 months after exposure in the first year of life, and (iii) long-term retention of a contingency is enhanced by as little as a few minutes of reactivation of the contingency. Interestingly such reactivation does not require practicing the contingency, but only perception of the stimulus involved in the contingency (e.g., seeing the mobile moving in a non-contingent manner) (e.g., Rovee-Collier et al., 1980). Thus, one might suggest that young infants create something like an “inverse model” of contingencies they are exposed to, since they are able to activate an entire contingency from its stimulus only. Moreover, it is worth noting that retention abilities of infants are enhanced when learning is spread over several days, suggesting that maybe, even in very young babies, sleep might help in consolidating sensorimotor learning (see Gómez and Edgin, 2015 for a review).

### Approaches in Robotics

Studies in artificial neural networks and robotics may shed some light on the memory processes illustrated above. Indeed, sensorimotor contingencies involve actions and outcomes that are perceived and encoded in the infants’ brain as, respectively, flows of motor commands (e.g., issued to the muscle fibers), and flows of sensory activations (e.g., the activations of retinal cones and rods). The contingency itself is encoded in brain neural networks as links between such input and output sensorimotor flows (this will be illustrated with an example in the Section on the “Blueprint architecture”). Neural networks thus represent good models to study the processes of memory involving contingencies such as those illustrated above.

A first contribution artificial neural networks can provide is to allow the specification of the mechanisms behind the memorization processes. Neural network studies on learning processes have shown that the ways in which neural networks can “memorize” experience are multiple and can be grouped into three broad categories depending on the source of the output to be associated to the (perceptual) input: supervised learning, unsupervised learning, and reinforcement learning. In supervised learning the output is provided to the agent by an external teacher or by another internal component (Bengio et al., 2017). In unsupervised learning the output is the result of algorithms that identify different possible statistical regularities in the sensory input (e.g., as in *self-organizing maps*, Kohonen, 2001). Finally, in trial-and-error learning processes, in particular reinforcement learning (Sutton and Barto, 2018), the output is actively searched for by the agent using active exploration of the environment and while attempting to maximize a reward measure with either an external or an internal origin. Goal babbling (Rolf et al., 2010) and goal-based self-generation (Santucci et al., 2016; Nair et al., 2018) are other examples where the learning of new actions pivots on the use, discovery, or generation of goals. Among the three classes, trial-and-error learning is clearly closer to contingency-based learning since it captures the fact that learning relies on free exploration of the environment and since contingent events would seem to be eligible to produce the reward signals needed to guide the learning process. These features are indeed at the core of the blueprint architecture presented below.

Once a memory is formed, it may undergo forgetting processes. One might wonder if the memory decay observed in infants is detrimental or advantageous for cognitive processing. Computational studies offer hypotheses for both cases. One explanation of memory loss might be that when new experiences are acquired, these might impair the existing memories deriving from previous experiences (“catastrophic interference,” French, 1999). The fact that robustness of infants’ memories differs at different ages might reflect the fact that initially their memories are stored in a broader overlapping unspecific way that makes them more vulnerable to interference from new experiences. In contrast, at older ages memories may be stored in a more specific way and so might be more robust to interference and hence appear to last longer in the developmental psychology tests (we are not aware of any model studying this). Reactivation experiments are subtler to explain. They might indeed involve complex re-consolidation processes that allow episodic memories (e.g., stored in hippocampus) to be transferred to semantic memories (in particular stored in cortex). These processes might allow stimuli perceived sometime after the main training to recall and cause consolidation processes that might later be manifested as enhanced memory. Some aspects of these processes are reproduced in computational models (e.g., Wei et al., 2016), but their nature is still elusive and largely not understood from a computational point of view.

## Generalization: To What Extent Is the Agent Capable of Generalizing a Sensorimotor Contingency to a New Situation?

Imagine now that someone had changed the carpet of the baby gym from red to green. How is the agent’s behavior affected by this change in the background context? Is the agent able to generalize its prior learning? And what if the objects themselves in the gym had been changed, instead of the background color?

### Evidence From Developmental Psychology

Generalization within the framework of sensorimotor contingencies would be the ability to transfer a contingency learned in one, original, set of conditions to a new situation. Experiments designed to test this have considered cases when the new situation differs from the original situation by a change in the stimulus (mobile) and/or in the context (color of the crib or testing room).

#### Changes in Stimulus

The effect of changing the stimulus was extensively investigated during the late 60 and 70’s using the non-nutritive sucking paradigm in which sucking of a pacifier produces a contingent feedback (visual or auditory). At that time, researchers were interested in infants’ discrimination abilities. They noticed that in non-nutritive sucking experiments, infants progressively lost interest in the visual or auditory stimulus generated by their sucking (this is known as habituation, see for ex. Aslin, 2007 for a review on visual habituation). Interestingly, if the stimulus was then changed to a new stimulus, infants’ interest increased, as reflected by an increase in their sucking rate. Results from these experiments suggest that infants are able to generalize their learning of contingency to a new stimulus when the change occurs immediately after learning (e.g., Milewski and Siqueland, 1975).

The effect of changing the stimulus was also explored by Rovee-Collier and her collaborators in 2- to 12-month-old infants using the mobile paradigm (see above for description) (see Hartshorn et al., 1998 or Rovee-Collier, 1999 for reviews). They observed that young infants — 2 to 6 months of age — could not transfer their learning of a contingency to a novel stimulus (i.e., a different mobile) when they were tested 1 day after training with this new stimulus. In contrast, older infants — 9 and 12 months of age — could easily transfer the learning of a contingency between a lever and the movements of a toy train to a novel train when they were tested on the next day and up to 2 weeks later but not for longer delays (3 to 8 weeks). Rovee-Collier’s team (Butler and Rovee-Collier, 1989) also tested generalization abilities of infants when both stimulus and context were changed simultaneously. They found that in this case infants were not able to generalize their learning even when they were tested only 1 day after training. This result is in line with what had been found previously — i.e., changes in stimulus strongly impair generalization abilities. Moreover, Rovee-Collier and her collaborators (Fagen and Rovee, 1976; Mast et al., 1980) explored the ability of 3-month-old infants to generalize their learning when quantitative aspects of the contingency were changed. They trained infants with one mobile and then switched to another mobile that provided weaker or stronger visual feedback (they did this by changing the number of components of the mobile). They found that even though infants noticed the change and reacted negatively (increase in negative vocalizations and cries and decrease in visual attention), they were still able to generalize their learning and even to adapt their kicking rate to maintain the original amount of visual feedback.

These studies show that younger infants have greater difficulty transferring their learning to a novel situation compared to older infants. But the question then arises of exactly what degree of difference makes for a “novel” situation? It seems that for young infants, very little change produces a “novel” situation. For instance, in the mobile paradigm, young infants were not able to generalize their learning as soon as a few components (one to three out of five) or the symbols painted on the mobile were changed (e.g., Fagen et al., 1976; Hayne et al., 1986; Adler and Rovee-Collier, 1994). Nevertheless, Rovee-Collier and her collaborators found four specific situations in which infants were able to generalize their learning to a novel stimulus (see Hartshorn et al., 1998 for a review). The first situation is when the novel and the original mobile share some visual features to which the infant selectively attends. The second situation is when the infant has forgotten the specific details of the original mobile. The third situation is when the infant is trained with a series of different mobiles. The fourth situation is when the infant is exposed to a novel object in motion after training is completed.

#### Changes in Context

Regarding the effect of changing the environment (e.g., testing room, color of the infant’s crib), Rovee-Collier and her collaborators (see Hartshorn et al., 1998 or Rovee-Collier, 1999 for reviews) studied this on infants aged 3 to 18 months of age. They showed that when tested 1 day after 2 or 3 days of training in the same context, both young infants — 3 to 6 months of age — and older infants — 9 to 12 months of age — were able to transfer their learning to a new context. This ability remains stable for delays longer than 1 day, and up to 1 month for older infants (9–12 months) but not for young infants (3–6 months). Thus, it seems that young infants are strongly sensitive to the context in which they learned a contingency. Moreover, what is considered as context in the infant’s learning is not only nearby and very salient cues but also the more global environment. For example, Hayne et al. (1991) trained 3-month-old infants in a portable crib in their bedroom, and then tested the infants in the same crib with the same mobile but in a different room. They found that infants did not show retrieval of the contingency when they were tested in a different room. Hartshorn and Rovee-Collier (1997) found the same results with 6-month-old infants using the train task. Nonetheless, as for a change in mobile, the failure in generalizing learning to a new context can be overcome by training in multiple contexts (e.g., Rovee-Collier and Dufault, 1991).

#### Conclusion From Developmental Psychology

It appears that over the course of development infants’ ability to generalize from one contingency to another increases gradually. While at first learning of sensorimotor contingencies is strongly impacted by the specific features of the contingent stimulus and by the context, learning becomes progressively more tolerant to stimulus and context changes during infants’ first year of life. This tolerance does not correspond to a decrease in infants’ ability to discriminate between the original and the new stimulus/context. Rather, it may reflect an increase in infants’ ability to recognize two situations as functionally equivalent and then to apply what they have previously learned in one situation to another one. Interestingly, this ability seems to emerge at the time when infants become self-locomoting (around 9–12 months of age with crawling and then walking), suggesting that infants become able to separate what has been learnt from the associated details/context only when they become able to spatially explore the environment by themselves (Rovee-Collier, 1996; Herbert et al., 2007).

### Approaches in Robotics

Generalization, the ability to respond to patterns and situations different from those previously experienced, is an appealing feature of neural-network models (McClelland et al., 1986), in particular when compared to “symbolic systems” (Russell and Norvig, 2010). Generalization ranges from the ability to keep responding when the main stimulus is slightly novel to the ability to keep responding when both the main stimulus and the context are very different. Regarding generalization over stimuli, research with computational models explains some of the observations of Rovee-Collier and colleagues on infants. In particular, the fact that the exposure to a large number of stimuli facilitates generalization is in line with one of the most important principles of neural networks, according to which increasing the size of the dataset used to train networks greatly benefits their generalization capabilities (Bengio et al., 2017). The reason for this is that important information tends to be present in all stimuli and so to be encoded, whereas irrelevant information due to noise or secondary features is much more variable and so tends to change connection weights in opposing directions and thus to not be encoded. With few experiences of stimuli, this mechanism is impaired as both important and irrelevant information is encoded thus resulting in poor generalization. Such mechanisms can presumably explain results observed using the mobile paradigm showing how infants generalize from an original mobile to another mobile with different elements (e.g., Rovee-Collier and Sullivan, 1980).

The design of neural models having generalization capabilities that are robust with respect to substantial stimulus/context changes is an important investigation known as *transfer learning*. This can involve either perceptual processes (Pan and Yang, 2010) or motor processes (Taylor and Stone, 2009; Tommasino et al., 2016). Transfer problems involve situations where the system learns some “source tasks” and has the opportunity to use the gained knowledge to solve new “target tasks.” The possibility of knowledge transfer depends on the similarity between the context and stimuli of the source and target tasks, and higher similarity enhances transfer. Such “similarity” however depends on the level of abstraction with which the context and stimuli are internally represented, in particular higher abstraction implies higher similarity. This might explain why older infants have an enhanced capacity to reuse acquired knowledge in novel situations: younger infants perceive reality through low-level features, so new situations appear to them as completely novel. On the other hand, older infants perceive reality through higher-level, more abstract features that make situations memorable with already acquired knowledge and skills.

If we look at the skills level, approaches that generate sequences of complex policies leveraging on some sort of “building block” skills might be seen as examples of generalization over motor policies, or better as examples of adaptation and transfer of knowledge between different contexts. In the option framework (Sutton et al., 1999) autonomous skill chaining has been tested in simple scenarios (e.g., Konidaris and Barto, 2009) and in robotic scenarios (e.g., Konidaris et al., 2012). Chunking of “lower-level” skills is used also within the imitation learning framework, where the presence of a cooperative tutor helps the artificial agent to gather new motor knowledge and link it together to generate more complex behaviors (Ugur et al., 2015; Duminy et al., 2018).

A trend in the study of artificial neural networks involves the proposal of systems having increasing ability to disentangle elements of the scene (e.g., objects) from the context. Work on these systems offers possible interpretations of the fact that infants’ capacity to recall knowledge on contingencies in different contexts increases with age. Generally, neural networks and robots engaged in recognizing objects process the whole scene. Currently the most advanced systems that do this, convolutional neural networks, automatically parse the image into regular sub-parts (local fields) and repeat the same processing on each of them (Bengio et al., 2017). Recently, the literature has proposed “regional convolutional neural networks” that do not process all parts of the image regularly, but actively focus on a small number of them that might provide richer information, thus leading to improved performance (see Maiettini et al., 2018 for a review). This process (e.g., for object recognition) is analogous to overt attention that in humans directs the gaze on salient areas of the scene (Marraffa et al., 2012; Wang et al., 2012). Here generalization is explained by the fact that irrelevant aspects of the scene are ignored altogether (Ognibene and Baldassarre, 2014). Compatible with these ideas, it could be that, at an early age, infants process the whole scene without being able to isolate single elements of it. With development, they might then develop the capacity to focus on limited portions of the scene so that the perception and hence memorization of contingencies is more effective (in parallel, memory structures might themselves become more robust).

These data and hypotheses on generalization (and transfer) capabilities coming from the computational literature might suggest new experiments with children. In particular, a test could check how babies at different ages learn to interact with objects and recall the acquired knowledge given different experimental conditions: one where the child has only one object to interact with, and another where the same object is surrounded by others (that could be within the workspace of the child or not). The hypothesis is that younger infants will be better able to recall the acquired knowledge when learning in a situation where no background/distracting objects are present, while older babies should be able to abstract from the context and transfer motor skills even when learning has occurred in a “crowded” setup. These are just examples of connections that could potentially be made between robotic models and empirical work.

## Goal-Directedness: Is the Agent Able to Use Sensorimotor Contingencies in Order to Attain Desired States?

When the agent (robot or baby) is exploring its baby gym, how does it decide what to do next? Does the agent *have in mind* a nice sound that it would like to hear? If yes, does this representation of the sound prompt the agent to touch the bell? Or on the contrary, does the agent need to actually see the bell again in order to touch it and produce the sound? In other words, are the agent’s actions goal-directed?

### Evidence From Developmental Psychology

The first issue in answering these questions resides in the definition of goal-directedness (see Kenward et al., 2009 for more detail on different views of goal-directedness). In developmental psychology, goal-directed actions are frequently described with respect to motor planning. An action is considered as goal-directed if the motor sequence is organized with respect to an object, an agent, a gesture or a posture (von Hofsten, 2004). With this definition, goal-directed actions would seem to be already present in very young infants (see for ex. Rochat and Striano, 2000; von Hofsten, 2004). In what follows here, we will characterize goal-directedness with respect to *decision-making* (Dolan and Dayan, 2013). More precisely, we will adopt the framework developed by Baldassarre and his collaborators (Santucci et al., 2016), where a *goal* is defined as a specific state of the world, and a goal-directed action is defined as an action made by an agent to attain this specific state of the world. Hence, to make a goal-directed action, the agent needs to possess two abilities: the ability to select a goal (i.e., a desired state of the world) and the ability to make the action that gives rise to the selected state of the world in the current context. This second ability implies that the agent has previously learned the contingent link between the selected goal and the action. One might say that the agent must have learned the “inverse model” of the contingency with regard to the context. Tackling the question of goal-directedness with respect to decision-making in infancy is challenging because cognitively demanding paradigms from the decision-making literature cannot be used (see Dolan and Dayan, 2013 for a review of these paradigms and Decker et al., 2016 for an attempt of implementation in a child experiment). Nevertheless, developmental psychologists have developed several strategies to explore goal-directedness: (i) means-end behaviors, (ii) anticipation abilities, (iii) bidirectionality of contingency, and (iv) “representation” of the action-outcome coupling. Exceptionally in this section we will discuss data involving infants older than 1 year as a reference for comparison to goal-directed behavior in younger infants.

#### Means-End Behaviors

A first way researchers have studied goal-directed actions in infants was by looking at infants’ ability to act on an object when this generates an effect on a second object — for example, pulling a support/string to retrieve an out-of-reach object or pushing a button to activate a device (e.g., Munakata et al., 1997). Such abilities are referred to as “means-end behaviors.” Infants are for example reported to solve the simplest tasks, such as selecting a lever, around 5–6 months of age (e.g., Lobo and Galloway, 2008) and to discover the pulling effect around 7–9 months of age (e.g., Munakata et al., 1997; Willatts, 1999; Babik et al., 2018). Many developmental psychologists (e.g., Piaget, 1936/1952; Diamond, 1991; Willatts, 1999) consider these early means-end behaviors as evidence for “intentionality” and goal-directedness in young infants. Nevertheless, as mentioned previously, certain apparent means-end behaviors could actually simply be stimulus-driven, meaning that the infants associate the view of the object with the pulling/pushing action without the notion of goal being involved (reinforcement learning). One method used to overcome this ambiguity is to offer the infant a choice between a set of potential “means” objects, among which only one allows the “end” object to be retrieved (e.g., a set of strings in which only one is attached to the out-of-reach object). With this protocol, it seems that young infants have great difficulties in choosing the effective “means” object, and if one considers that solving such a multiple means-end problem is a proof of goal-directedness, it seems that goal-directedness is only acquired during the second year of life (e.g., Rat-Fischer et al., 2014). The acquisition of goal-directedness has also been studied through investigation of the baby’s understanding of other people’s means-end behaviors (e.g., Carpenter et al., 2005; see Elsner, 2007 for a review). Some authors have argued that babies have the ability to perceive other people’s actions as *intentional* from an early age, and that they are able to use this early *teleological reasoning* to selectively imitate other people’s actions (e.g., Gergely et al., 2002). However, some authors have questioned the precocity of babies’ ability to perceive the actions of others as *intentional* (e.g., Paulus et al., 2013) and have argued that this ability develops gradually during the first years of life as a function of the baby’s sensorimotor experience (e.g., Sommerville et al., 2005) — see Paulus and Király, 2013 for a review.

#### Anticipation Abilities

Going a step further in the study of goal-directedness, developmental psychologists have investigated infants’ ability to anticipate the appearance of a contingent feedback that follows after realizing an action. Wang et al. (2012) explored this question on 6- and 8-month-old infants using a gaze contingency set-up. In their experiments, infants could activate the appearance of a picture in the middle of the screen by looking at a red disk located on one side of the screen. The authors measured the extent to which the infant anticipated the appearance of the picture — namely if the infant started to look at the middle of the screen directly after looking at the contingent disk but before the appearance of the picture. They found that at both ages, infants were able to learn the contingency to anticipate the contingent feedback. Along the same lines, Kenward (2010) found that 10-month-old infants were able to anticipate the appearance of a video generated by pushing a button. Adopting the assumption that anticipation of an action’s effect means that this action was goal-directed, one might consider that infants are able to make goal-directed actions from at least 6 months of age. Nevertheless, anticipation paradigms do not guarantee that infants are capable of *goal selection*. Indeed, one might argue that when an infant anticipates the consequences of its actions, this does not mean that the infant has selected this action to obtain a desired goal (contingent feedback). Instead, the infant could have learnt the contingency by reinforcement learning without any use of the notion of goal. With the aim of obviating this weakness of anticipation paradigms, developmental psychologists have used a paradigm coming from the animal literature and more effectively testing goal-directedness: the revaluation paradigm (see later). But before addressing this issue, a simpler question to ask is: to what extent is infants’ sensitivity to sensorimotor contingencies bidirectional?

#### Bidirectionality of Contingency Learning

It has been shown in adults that when a subject has learned a contingency between an action and an effect, the simple presentation of the effect activates the action involved in the previously learned contingency (see, e.g., Elsner and Hommel, 2001). This bidirectionality of contingency learning has been defined as one of the building blocks of goal-oriented actions (Verschoor and Hommel, 2017), since it ensures that a subject is able to identify the appropriate action to achieve an effect. Infants’ ability to identify an action from its effects has mainly been investigated by Verschoor et al. (2010, 2013). In Verschoor et al. (2010), the authors first exposed 9-, 12-, and 18-month-old infants to two sounds: one sound was self-produced by the infant by touching a panel and the other sound was independent of the infant’s behavior. Then, the authors measured infants’ propensity to realize the contingent action (i.e., touch the panel) after presentation of the contingent or the non-contingent sound. The authors found that infants at all ages were faster to produce the contingent action in response to the contingent sound than to the non-contingent sound, suggesting that from 9 months of age, infants are able to identify an action from its effects. In their second study (Verschoor et al., 2013), the authors exposed 7-, 12-month-old infants (and adults) to two contingencies, each associating respectively a right and a left saccade to a different sound. Then, they measured participants propensity to make the “good” contingent action (right or left saccade) after being exposed to its previously learnt effect (one of the two sounds). They found that the older infants (12 months of age) and adult were able to identify an action from its effect whereas younger infants (7 months of age) were not able to do so. Interestingly, they found that both 7- and 12-month-old infants were surprised (shown by an increase in pupil dilation) when they made the “wrong” action, suggesting that 7-month-old infants formed bidirectional knowledge of the contingencies but were not able to use this knowledge in action selection.

#### “Representation” of the Action-Outcome Coupling

The studies presented above suggested that after 9 months of age, infants are able to “predict” the consequences of their actions and to identify an action from its effects. However, one question remained open: are infants able to represent a desired effect *in their mind*, and to make an action in order to obtain this effect? To investigate this question, developmental psychologists have used the revaluation paradigm. This paradigm works as follows. In a first phase (the acquisition phase), the infant learns two action-effect couples. Then, the value of one of the contingent effects is changed. After this relative change in valuation of one of the stimuli, it is assumed that the infant has a strong preference for the non-devalued contingent effect. At the end (test phase), the infant has the opportunity to execute the previously learnt contingent actions to obtain the associated effects. It is important to highlight that the contingent feedback is not displayed at this phase of the experiment in order to ensure that the infant’s choice of action is goal-directed and is not stimulus-driven. It is considered that an infant is capable of goal-oriented action if she or he prefers to perform the action involving non-devalued feedback in comparison with the other action. Indeed, such behavior suggests that (i) the infant is able to generate a goal (here the non-devalued stimulus) and (ii) the infant is able to select the action that leads to this goal, without any stimulus being presented. Klossek et al. (2008) tested to what extent infants’ sensitivity to the revaluation paradigm evolves in the second and third year of life. They tested three groups of infants: 16 to 27, 27 to 37, and 37 to 48 months of age. All age groups were able to learn two contingencies in the acquisition phase and were sensitive to revaluation (i.e., inducing a preference for one of the stimuli generated by the contingent actions). But, interestingly, in a test phase, only the two older groups of infants, from 27 to 48 months of age, were able to show their preference in stimulus (goal selection) by making the correct action (action selection). This last result means that infants younger than 2 years of age were not able to make goal-directed actions, and remained predominantly stimulus-driven in their behaviors. To ensure that this inability to make goal-directed actions in younger infants was not due to the complexity of the task, Kenward et al. (2009) developed a simplified version of the revaluation paradigm. In their setup, the infants had to learn only one contingency in the acquisition phase. Then, for one group of infants (the revaluation group) the stimulus used as contingent feedback in the acquisition phase was increased in value, while a control group were occupied by an independent irrelevant task. In the test phase, infants’ propensity to repeat the contingent action in the absence of feedback was compared between the revaluation and control groups. The authors tested infants of three ages 14, 19, and 24 months. Here again, the authors found that infants at all ages were able to learn the contingency in the acquisition phase and were sensitive to the revaluation. But the authors observed that only at the oldest age (24 months), did infants trained in the “revaluation group” show a higher number of contingent actions during test compared to the control group. If one accepts that the revaluation paradigm is a test of goal-directedness, it seems that infants only become able to use goals to select their actions after 2 years of age.

#### Conclusion From Developmental Psychology

Taken together these studies suggest that infants’ ability to make goal-directed actions emerges progressively during the first 3 years of life. Young infants (before approximately 9 months of age) seem sensitive to sensorimotor contingencies, and even seem able to predict the consequences of their actions, but without being able to use this knowledge in order to select their actions. Thus, they are capable of goal selection (since they can develop a preference for a stimulus) but not of action selection. Then, at the end of their first year of life, infants become able to select their actions in a stimulus-driven fashion, since they can recall an action when they first perceive the stimulus (e.g., move to make a static mobile move) but not when they have to cause presentation of the desired stimulus (e.g., obtain a specific sound). Finally, at the end of their second year of life, infants become able to select an action in order to attain a desired (and not necessarily currently perceived) state of the world. Thus, one might conclude that it is only at this age that infants become really capable of goal-directed actions. Note that this progressive pattern of acquisition might possibly be related to the progressive acquisition of inhibitory abilities in infancy.

### Approaches in Robotics

Computational modeling confirms that observing anticipatory behavior like that observed in the gaze contingency experiments of Wang et al. (2012) does not imply that the infants are goal-directed. In this respect, Marraffa et al. (2012) presented a model that mimics the behavior of the infants when tested in a simulated version of the set-up used in the experiment of Wang and colleagues. Like the infants, the model learns by reinforcement learning to gaze at salient parts of a (simulated) screen to cause the appearance of a rewarding image. The model incorporates bottom–up and top–down attention mechanisms that allow it to learn as fast as real infants. The model also exhibits the same anticipatory gazing behavior as the infants. This is explained by the fact that with learning as soon as the simulated infant sees the cue associated with the appearance of the image, it immediately looks at the place where the image will appear as this is the place to look at to receive the reward. Of course, this only shows that reactive behavior and reinforcement learning are sufficient to produce the anticipatory behavior seen in infants, but does not rule out the possibility that they rely on goal-directed mechanisms. Regarding the experiments of Verschoor et al. (2010, 2013) on the bidirectionality of contingency learning, in future work it would be interesting to check which mechanisms are needed to reproduce the observed behaviors and whether they represent a precursor of more sophisticated goal-directed processes.

While goals are a fundamental element in classic artificial intelligence planning systems (Russell and Norvig, 2010), their use in robotic and machine learning neural-network systems has been limited by the fact that the focus is usually on the solution of only one user assigned task. In this context, goals have been used only recently to face situations involving multiple tasks where robots need to decide on which of them to focus (Hart and Grupen, 2011; Santucci et al., 2013a). In particular, in developmental robotics goals are crucial to support the learning of autonomous agents as they allow them to decide on which task to focus on the basis of intrinsic motivations (Santucci et al., 2012). Moreover, goals are also important to explore the task space (Cartoni and Baldassarre, 2018; Nair et al., 2018) and, as described in the framework of goal babbling (Rolf et al., 2010), they can possibly contribute to reduce this space thereby avoiding the “curse of dimensionality” problem (Bishop, 1995). On this basis, different artificial agents have been proposed that are able to autonomously discover and select their own goals and use them to drive the learning process (e.g., Baranes and Oudeyer, 2013; Seepanomwan et al., 2017). While it can be complicated to identify goal-directed processes in very young babies, robotic systems such as GRAIL (Santucci et al., 2016) are endowed with the possibility of storing and explicitly selecting interesting possible environment states, thus turning them into goals that can determine action selection and skill learning. These robotic models suggest that the possibility of performing goal-directed behavior should go hand-in-hand with the possibility of forming representations of objects/states/events that can be reactivated in the absence of corresponding entities in the world. This allows an agent to act so as to increase the chance of obtaining “desired outcomes” (goals). This ability has to be paired to a “goal-matching” ability that compares the actual state of the world with the pursued goal so as to detect when it is achieved: this mechanism is a possible way to computationally operationalize how contingency detection might guide the acquisition of skills, as illustrated in detail in the following section.

## Blueprint Architecture: A Theoretical Integration of the Elements of Contingency-Based Learning

### The Elements and Functioning of the Blueprint Architecture

**FIGURE 1:**
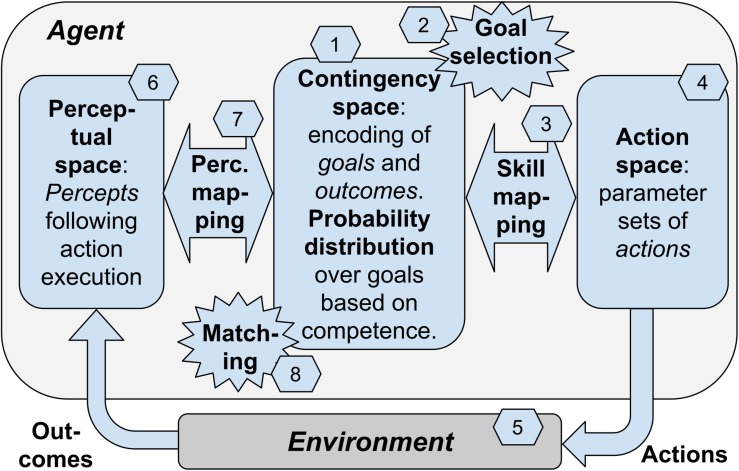
The blueprint architecture incorporating our hypothesis about the key elements underlying open-ended learning of multiple skills. Boxes: the components of the architecture. Numbers: sequence of processes happening in one trial of functioning of the system.

In the previous sections we have considered multiple elements involved in contingency-based learning. We now consider a “blueprint architecture” representing a theoretical scheme that integrates these elements in a whole framework (Figure 1). A first version of the architecture was proposed in Baldassarre et al. (2018). The architecture proposes a possible specification of the contingency-based learning mechanisms and processes considered this far, and also adds to them further mechanisms needed to have a fully autonomous agent. The value of the architecture is that it proposes a possible way to integrate such processes, thus offering a coherent view of how they could work together. The architecture can also inform the construction of specific computational models, as was done in Mannella et al. (2018).

The architecture is based on the hypothesis that contingency-based learning encompasses general mechanisms able to support the self-generation of goals, the encoding of the perceptual consequences of actions, and the learning of actions of any level of complexity, e.g., from moving a single finger to performing grasping with the whole hand. To illustrate the architecture, we will focus on a case involving learning to reach parts of the agent’s own body, a relevant form of knowledge that infants acquire soon after birth. However, we hypothesize that analogous contingency-based learning processes might support the acquisition of more sophisticated classes of goals and actions.

A key idea at the core of the architecture is that contingency-based learning relies on a specific type of events that are *internal* to the agent and reflect the sensorimotor contingencies happening in the environment, i.e., the motor action performed by the body and the physical consequences that it causes in the environment. These events, called “matching events,” correspond to a match between the selected goal encoding (encoding of the desired perceptual effect of the action), and the outcome encoding (encoding of the actual perceptual effect of the action). For example, intrinsic motivation might lead an infant to select the goal of touching a part of her chest. The goal activation might trigger the performance of an action directed to accomplish it. The performed action leads to a perceived effect in the environment (e.g., feeling of body touch). The perceived effect is internally represented in the brain and may or may not match the representation of the pursued goal. In case of “success” (the action produced the desired outcome), the matching event plays a crucial role in the architecture as it contributes (a) to modify the intrinsic motivation that leads to goal-selection, and (b) to guide the learning of perceptual mapping, the learning of the skill mapping, and a learning process leading to align the goal encoding and the outcome encoding within the contingency space. We now review these processes in more detail.

The architecture consists in a number of components that might be implemented in different ways. The components are linked between them to exchange information and learning signals and they tightly work together to support the whole functioning of the system. However, it is useful to distinguish between them as this facilitates the explanation and also facilitates specific implementations. As mentioned above, an instance of the blueprint architecture has been specifically implemented in Mannella et al. (2018). The model considers a condition where an infant performs exploratory movements and this allows her to learn to touch own parts of her own body when desired (e.g., to scratch and remove undesired stimuli). The model represents the body of the agent with a simple simulated kinematic planar robot formed by a torso and two arms: the “torso” is represented by a segment and each arm is represented by three linked segments attached to the two ends of the torso and linked by motorized joints (the agent’s body has thus has 6 degrees -of -freedom in total). The body has a number of touch sensors along the torso and the arms; these sensors can be activated by the touch of the two agent’s “hands” (the ends of the arm links). The input to the model is formed by the activation of the touch sensors and its output is formed by commands issued to the six motors of the two arms. We now illustrate the components of the blueprint architecture, and at the same time we furnish examples of how they were instantiated in Mannella et al. (2018):

(a) *Contingency space*: a set of vectors (e.g., related to the possible activations of a group of neurons; the vectors are the points forming the space) each of which can represent two things: (a1) a goal encoding the desired perceptual effect of the action triggered by the goal (where the “perceptual effect” can involve proprioception, perception of the outer world, or both); (a2) the encoding of the actual effect resulting from the performance of an action. Importantly, as discussed below, with learning the system aims to progressively drive each goal (encoded in the contingency space) closer to the outcome (as encoded in the contingency space) of the action triggered by the goal. The contingency space can be either discrete or continuous. In the model instantiation, the contingency space is 2D and is represented by a map of the output units of a “self-organized map” model (SOM; Kohonen, 2001); each of these units can represent: (i) a goal encoding the *desired* perceptual effect of the action triggered by the goal, where the “perceptual effect” involves the activation of the touch sensors; (ii) the encoding of the *actual* effect resulting from the performance of an action directed to pursue the goal. In this case, the contingency space is represented in a discrete fashion, so the agent can form a number of goals equal to the number of units of the SOM.
(b) *Goals*: points of the contingency space that represent *desired* perceptual effects of actions, and can be internally selected and activated to trigger the related action. In the model instantiation, a goal is encoded by a unit of the SOM through its connection weights linking the input layer of the system (that is also the input layer of the SOM) and the contingency-space units; each goal represents the *desired* touch-sensor activation of the action linked to the goal and encoded in the motor mapping.
(c) *Goal probability distribution*: a probability distribution over goals, used to select the goal to pursue, and that depends on intrinsic motivations. In the model instantiation, the SOM units have a probability distribution over them, used to select the goal to pursue and triggering the related action; the probability distribution depends on a competence-based intrinsic motivation (see below).
(d) *Skill mapping*: mapping from the selected goal within the contingency space, to the action directed to pursue it. In the model instantiation, this mapping is represented by an “echo-state network” (ESN; Jaeger, 2001), a dynamical model where the input (goal) leads to produce output values that change in time and encode the sequence of commands issued to the joints of the arms which so produce a movement trajectory possibly leading to touch the body with a hand.
(e) *Action space*: set of action parameters where one parameter combination represents one action that can be selected by the skill mapping. In the model instantiation, this is the set of possible movement trajectories encoded in the parameters of the ESN: the performance of a trajectory can be triggered by the selection of the related goal.
(f) *Perception space*: set of sensor encodings of the possible effects (outcomes) caused by actions in the environment. In the model instantiation, this is the set of sensor encodings corresponding to the possible effects (outcomes corresponding to the possible activation patterns of the touch sensors) caused by actions in the environment.
(g) *Perception mapping*: mapping from the percepts resulting from action performance to their encoding within the contingency space. In the model instantiation, this is the mapping implemented by the SOM from the percepts resulting from action performance (activation of the touch sensors) to their encoding within the contingency space (SOM output units).

The architecture works and learns in trials, and for each trial the following processes take place (see Figure 1, numbers in hexagons):

(1) Initially the skill mapping and the perception mapping are randomly initialized so the points in the contingency space have random correspondences to the actions encoded in the action space and to percepts encoded in the perceptual space. In the model instantiation, these random initialization involve the connection weights of the skill mapping (ESN) and the perception mapping (SOM).
(2) At the beginning of each trial the agent selects and activates a goal in the contingency space on the basis of a certain probability distribution explained below. In the model instantiation, the probability distribution is used to select a goal represented by a unit of the SOM output layer.
(3) The skill mapping generates an action parameter set encoded in the action space. In the model instantiation, the ESN generates an action, i.e., a movement trajectory of the two arms, lasting for a given time.
(4) The agent performs in the environment the action corresponding to the selected parameter set. In the model instantiation, the action is performed with the two arms.
(5) As an effect of the action, the environment changes into a certain state (outcome). In the model instantiation, this is represented by the activation of the touch sensors.
(6) The outcome is encoded as a point in the perceptual space. In the model instantiation, the activation of sensors is mapped onto the SOM output units by their connection weights (perceptual mapping).
(7) The point in the perceptual space corresponding to the action outcome is encoded into a point in the contingency space that constitutes the internal representation of the outcome. In the model instantiation, the most active unit of the SOM is considered as the internal representation of the outcome within the contingency space.
(8) The internal outcome representation can match the goal or not (if the contingency space is discrete), or it can have a certain degree of similarity with it (if the contingency space is continuous). This allows the computation of a matching signal: this signal has a discrete 0/1 value in the case the contingency space is discrete, or a continuous value within [0, 1] in the case the contingency space is continuous (in this case it is computed on the basis of a suitable outcome/goal metric). In the model instantiation, the internal outcome representation can match the initially selected goal or not (the contingency space is discrete) and this produces a 0/1 “matching signal.”

The matching value is used to determine the probability distribution over the goals that supports the selection of the goal to pursue (number 2 in the figure). The probability distribution is generated on the basis of intrinsic motivations, for example in the model instantiation these are related to competence improvement (the goal with the highest competence improvement has a highest probability of selection; Santucci et al., 2013b).

The matching value is also used as a learning signal to drive the learning processes involving the perceptual mapping and the skill mapping. In particular, it is used as a reward to: (a) find the action parameters by reinforcement learning, and use them to update the skill mapping (the ESN in the model instantiation); (b) update the perceptual mapping so that the percepts that are similar in the perceptual mapping connect to similar points in the contingency space (training of the SOM in the model instantiation); (c) the perceptual mapping is also updated, in parallel to the previous learning ‘b,’ so that the internal outcome representation matches, or becomes more similar, to the goal representation; the skill mapping is also updated, in parallel with the previous learning ‘a,’ to improve such matching; the perceptual mapping and the skill mapping are hence updated subject to two objectives, namely one directed to improve the outcome-goal matching and one directed to improve the outcome representations and the actions to pursue the goals (technically, the objective function used for each training has two components). Through these learning processes, the internal outcome representation and the goal representation tend to align, i.e., each goal tends to produce an action that causes an outcome in the world that once perceived is encoded in a point in the perceptual space that matches the goal. The agent thus discovers the possible outcomes that it can produce in the environment, learns to encode them in the contingency space, and acquires the motor capabilities needed to accomplish such outcomes when desired.

In the model instantiation, we have considered here a scenario where the system is interested in sensorimotor contingencies involving outcomes produced on the agent’s own body. Processes similar to those considered here might however be potentially used to produce contingencies involving the outer world, for example touching, moving and grasping objects. This would require a richer sensory input informing the agent about the outcomes that its action can produce on the environment, for example visual and acoustic sensors.

### The Blueprint Architecture as a Link Between Different Aspects of Contingency Learning, and Limitations

The blueprint architecture might be used to link the different issues regarding sensorimotor contingencies discussed in the previous sections and concerning learning of body-related sensorimotor contingencies, processes involving memory of contingencies, generalization to different contexts and learning of goal-directed contingencies. Goal-directedness represents the central element of the architecture. In particular, the contingency space of the architecture encodes goals, goals are selected to drive exploration, and goals gradually converge toward outcomes that the agent understands it can produce through its body and actions.

As mentioned, the goals and the related actions of the models implementing the blueprint architecture can involve the acquisition of knowledge on the agent’s own body or knowledge related to the outer world, e.g., about objects. In this respect, the blueprint architecture suggests that there might be common processes guiding the acquisition of knowledge on the body and on the outer world. Such processes might even support the learning of social contingencies (Gergely and Watson, 1999).

The blueprint architecture, and the possible models implementing it, have the function of storing information within the sensory mapping and the skill mapping, e.g., in the form of connection weights of the neural networks used to implement them. This allows the use of the models to investigate the processes involving the memories of contingencies studied in developmental psychology, for example retention and reactivation processes. Retention might in particular be studied by using computational protocols based on training-interference-recall tests (Caligiore et al., 2014b). Similarly, reactivation could possibly be studied based on the implementation in the models of mechanisms encompassing memory consolidation (Wei et al., 2016).

A last issue discussed here in relation to sensorimotor contingencies is generalization. Generalization is a major feature of neural networks (Bengio et al., 2017), so psychological experiments on context-dependent generalization of sensorimotor contingencies might be studied through models implementing the blueprint architecture with neural networks. This would require the encoding in input of context alongside the stimulus directly involved in the contingency.

The blueprint architecture has some limitations that became apparent during its construction and that might be faced by future empirical and modeling work. One limitation concerns the agent’s capacity to understand that a certain effect observed in the environment is caused by the agent’s own action and not by the spontaneous evolution of the environment or by other agents. In this respect, we have so far assumed that all the effects that the agent observes are caused by its own actions. This is a good assumption in some conditions (imagine an infant exploring her body while in the cradle) but not always. The capacity we are discussing is linked to *causality* and can be detected as the difference between the probability that a certain outcome happens after a certain action is performed and the probability that the outcome happens if the action is not performed (Gopnik et al., 2004). To check the effectiveness of actions, the architecture discussed above should be enhanced with suitable mechanisms to observe the environment multiple times after having executed an action or not, and to compare the probability of achieving the outcome in the two cases (for some computational models using mechanisms to face the problem of agency see: Pitti et al., 2009; Butko and Movellan, 2010; Sperati and Baldassarre, 2017).

Another very important issue not considered by the blueprint architecture is the fact that the actions produced in the environment can produce many different effects on the body and the environment. The agent should therefore be endowed with a mechanism to decide which effects (action-effect contingencies) are ‘interesting’ and so worth triggering the learning processes illustrated above. One possibility is that the agent should consider as interesting ‘effects’ corresponding to a *change* in the environment; the rationale of this is that actions causing a change in the body/environment might be useful in the future (Barto et al., 2004; Santucci et al., 2016; Sperati and Baldassarre, 2017). Another possibility is to use intrinsic motivations such as *novelty* or *surprise*, i.e., to consider as interesting those effects of actions that respectively have never/rarely been experienced before, or that violate expectations (Barto et al., 2013). A last possibility is that the agent considers as interesting those action-effects that might be useful to achieve further effects for which the given contingency is a precondition, in particular in conditions involving the production of action sequences (various techniques can be used to this purpose, e.g., see McGovern and Barto, 2001). Overall, these are important open issues at the core of development and open-ended learning in robotics that deserves further exploration in the future, and that might be open to empirical testing by developmental psychologists. A first step in this direction has indeed been made by Kidd et al. (2012) in experiments showing that 7- and 8-month-old infants learn contingencies that are of intermediate level of difficulty, given their current knowledge – the so-called “Goldilocks” effect.

## Conclusion and Perspectives

The sensitivity of an agent to the links between its actions and their consequences is central in the theories of psychology that roboticians use in their work, such as the sensorimotor contingency theory (e.g., O’Regan, 2011), the Gibsonian theory of affordances (Gibson, 1979) or the predictive coding theory (e.g., Clark, 2013). Sensitivity to sensorimotor contingencies is particularly important in developmental robotics because it provides a simple way to equip an agent with the ability to learn to interact with the world using self-organized exploration of its environment. In this paper, we have strengthened this statement by demonstrating how the use of this sensitivity by babies allows them to acquire and/or mature new motor and cognitive abilities such as body knowledge, memory, generalization and goal-directedness. In addition, our literature review highlights the links between conclusions obtained in developmental psychology and current work in robotics, and thus offers readers from robotics a concrete and direct application to their field of study.

The model proposed at the end of the article shows how exploitation of sensitivity to sensorimotor contingencies, combined with the notion of “goal” (i.e., a desired state of the world), allows an agent to organize its exploration of the world and develop new sensorimotor skills, in the present case the skills that underlie understanding of the structure of its body. This model is conceived to be suitable for all types of sensorimotor learning, and we hope that in the future it will be adapted both to better understand baby development and to build robots capable of more effective autonomous learning. In its present state our blueprint architecture is just an example of the kind of robotic modeling that could be done to make contact with empirical results from developmental psychology. The model has many arbitrary aspects and many imperfections, but hopefully it provides a first step in the direction of increasing contact between the two fields. More generally, the sections in the present article suggesting links between empirical results and concepts in developmental robotics are only first steps toward such links. We do think however that closer collaboration between developmental psychology and developmental robotics should help (i) to overcome the lack of mechanistic approaches in developmental psychology, and (ii) to provide new ideas and concrete data that roboticians can use to develop open-ended learning. We hope that our article contributes to the emergence of a common language between the two fields that will facilitate future fruitful collaboration.

## Author Contributions

LJ reviewed the literature in developmental psychology, and wrote the sections “Abstract,” “Introduction,” “Conclusion and Perspectives,” and the psychological parts. VS and GB reviewed the literature in robotics and wrote the robotics parts. JO’R reviewed the work critically and provided approval for the publication of the content.

## Conflict of Interest

The authors declare that the research was conducted in the absence of any commercial or financial relationships that could be construed as a potential conflict of interest.

## Footnotes

1 Nonetheless, even newborns are able to display distinctive actions when they are in specific conditions (Grenier, 1981; van der Meer et al., 1995, 1996; van der Meer, 1997).
